# *Paris polyphylla* var. *yunnanensis* Leaf-Derived Extracellular Vesicle-Like Particles Enhance Periodontal Regeneration

**DOI:** 10.34133/bmr.0291

**Published:** 2025-12-09

**Authors:** Guobin Huang, Anfan Liu, Yu Hu, Rongqiang Yang, Zichao Dai, Wei Meng, Yan Yan, Hefeng Yang, Song Li

**Affiliations:** ^1^ Yunnan Key Laboratory of Stomatology, Kunming Medical University School and Hospital of Stomatology, Kunming, Yunnan, PR China.; ^2^ Department of Dental Research, Kunming Medical University School and Hospital of Stomatology, Kunming, Yunnan, PR China.; ^3^ Outpatient Department, Kunming Medical University School and Hospital of Stomatology, Kunming, Yunnan, PR China.; ^4^ Department of Orthodontics, Yan’an Hospital Affiliated to Kunming Medical University, Kunming, Yunnan, PR China.; ^5^ Department of Stomatology, The First People’s Hospital of Kunming, Kunming, Yunnan, PR China.

## Abstract

Periodontitis, a highly prevalent chronic inflammatory disease globally, faces substantial challenges in achieving periodontal tissue regeneration, necessitating the development of novel therapeutic strategies. Chinese herbal medicine-derived extracellular vesicles (CHMEVs), natural nanoscale carriers enriched with bioactive components from medicinal plants, exhibit unique therapeutic advantages in tissue repair. Here, we isolated extracellular vesicle-like particles from *Paris polyphylla* var. *yunnanensis* leaves (PP-L-EVLPs), a traditional Chinese medicinal herb native to Yunnan, and systematically evaluated their therapeutic potential for periodontal regeneration. PP-L-EVLPs were efficiently internalized by periodontal ligament stem cells (PDLSCs), enhancing their proliferation, migration, and osteogenic differentiation through up-regulation of ALP, RUNX2, and OPN. PP-L-EVLPs significantly suppressed the protein expression levels of lipopolysaccharide-induced interleukin-6 (IL-6) and IL-8 in PDLSCs. In a rat alveolar bone defect model, PP-L-EVLPs significantly promoted bone regeneration, as evidenced by micro-computed tomography, histology, and immunohistochemistry. Biosafety evaluations revealed no histopathological abnormalities or genotoxicity in major organs of Sprague–Dawley rats treated with PP-L-EVLPs. This study is the first to confirm that PP-L-EVLPs exhibit cell migration-promoting, anti-inflammatory, and osteogenic activities with excellent biosafety, offering a novel natural nano-based therapeutic strategy for periodontitis treatment.

## Introduction

Periodontitis, ranked among the 6 most prevalent inflammatory diseases worldwide, exhibits a high incidence rate. According to the Fourth National Oral Health Epidemiological Survey, periodontitis affects over 70% of middle-aged and elderly individuals in China, with Yunnan Province exhibiting a notably higher prevalence than the national average [1,2]. As a chronic inflammatory disorder, periodontitis is initiated by dental plaque biofilm and progresses through dysregulation of periodontal cell homeostasis. Its pathological hallmarks include gingival bleeding, periodontal pocket formation, connective tissue destruction, and alveolar bone loss, ultimately leading to tooth mobility and exfoliation [3]. Under sustained chronic inflammation, immune dysregulation within the periodontal niche reduces the quantity and functional capacity of residual periodontal ligament stem cells (PDLSCs), resulting in severe microenvironmental disruption. This mechanism underpins the persistent nature of periodontitis and its progressive tissue destruction [4]. Current clinical interventions, primarily mechanical debridement and antibiotics, provide transient inflammatory control but lack the capacity to achieve periodontal tissue regeneration [3]. Consequently, the development of novel therapeutic strategies targeting microenvironmental restoration and stem cell-based regeneration is urgently warranted.

PDLSCs are the core effector cells for periodontal regeneration, but their osteogenic differentiation capacity is significantly suppressed in the inflammatory microenvironment of periodontitis [5]. Effectively regulating the osteogenic differentiation of PDLSCs is critically important for periodontitis treatment. Recent research on periodontitis therapies has focused on stem cell-based approaches, exogenous growth factor delivery, and mammalian-derived extracellular vesicles [6–8]. However, limitations such as restricted sources, low yield, immune rejection, high costs, and ethical controversies hinder their development and clinical application. Plant-derived extracellular vesicles (PDEVs), membrane-bound nanoparticles secreted by plant cells, share structural similarities with mammalian extracellular vesicles, consisting primarily of a lipid bilayer that encapsulates diverse proteins, nucleic acids, and bioactive molecules [9,10]. PDEVs exhibit the ability to enter mammalian cells and modulate cellular activities while also serving as drug carriers to enhance drug stability and cellular uptake [11,12]. Owing to their abundant sources, high yield, low immunogenicity, biocompatibility, simplicity, safety, eco-friendliness, low cost, and minimal toxicity, PDEVs have emerged as promising natural nanotherapeutics with unique advantages in tissue regeneration medicine [13].

*Paris polyphylla* var. *yunnanensis*, a plant of the Liliaceae family primarily distributed in Yunnan, Sichuan, and Guizhou provinces, serves as a key raw material for traditional Chinese medicines such as Yunnan Baiyao and Gongxuening. The *Pharmacopoeia of the People’s Republic of China* documents its efficacy in clearing heat and detoxifying, reducing swelling and relieving pain, and cooling the liver to calm convulsions, with clinical applications for sore throat, snake/insect bites, and traumatic injuries. Modern pharmacological studies confirm that its primary bioactive compounds, polyphyllins, exhibit anti-inflammatory, antioxidant, antimicrobial, and anticancer properties [14–16]. Specifically, polyphyllins inhibit the growth of microorganisms including *Pseudomonas aeruginosa*, *Staphylococcus aureus*, *Listeria innocua*, *Escherichia coli*, *Salmonella enterica*, and *Shigella sonnei*, suggesting their potential utility in treating oral diseases [16–18].

Expert Consensus on Research and Application of Chinese Herbal Medicine-derived Vesicles (2023 Edition) states that extracellular vesicles derived from Chinese herbal medicines contain the vast majority of bioactive components and represent the concentrated essence of herbal efficacy [19]. This study employed a novel method combining high-speed centrifugation with density gradient centrifugation to isolate extracellular vesicles from fresh *P. polyphylla* var. *yunnanensis* leaves. In vitro experiments demonstrated that extracellular vesicle-like particles derived from *P. polyphylla* leaves could be effectively internalized by PDLSCs and significantly promoted their osteogenic differentiation. In vivo, an alveolar bone defect model in Sprague–Dawley (SD) rats confirmed that these extracellular vesicle-like particles effectively enhanced the repair of bone defects. These findings suggest that PDEVs hold promising potential as a novel natural therapeutic pathway for periodontal tissue regeneration engineering.

## Materials and Methods

### PP-L-EVLPs isolation and purification

Fresh leaves of *P. polyphylla* var. *yunnanensis* were washed 3 times with deionized water, cut into fragments (5 mm^2^), and homogenized in phosphate-buffered saline (PBS) buffer (1:5, w/v) using a tissue homogenizer. The homogenate was filtered through sterile gauze to remove residual plant debris. The filtrate underwent sequential centrifugation at 4 °C: primary clarification at 500*g* for 10 min, secondary clarification at 2,000*g* for 20 min, and tertiary clarification at 12,000*g* for 30 min (repeated twice). Subsequent ultracentrifugation (120,000*g*, 120 min) yielded crude PP-L-EVLPs (extracellular vesicle-like particles from *Paris polyphylla* var. *yunnanensis* leaves) pellets, which were resuspended in PBS with vigorous vortex mixing.

A discontinuous gradient comprising 8%, 15%, 30%, 45%, and 60% (w/v) sucrose solutions was prepared in ultracentrifuge tubes. The resuspended crude extract was layered onto the gradient and centrifuged at 150,000*g* for 2 h. The 30% to 45% sucrose interface fraction containing PP-L-EVLPs was collected, diluted with PBS, and pelleted by ultracentrifugation (150,000*g*, 70 min). Final purification involved resuspension in PBS, filtration through 0.22-μm membrane filters, and storage at −80 °C. The protein concentration of purified PP-L-EVLPs was quantified using the bicinchoninic acid (BCA) protein quantification assay kit (Proteintech, USA).

### Characterization and purity assessment of PP-L-EVLPs

The morphology of PP-L-EVLPs was observed using transmission electron microscopy (TEM). Nanoparticle tracking analysis (NTA) was employed to determine the nanoparticle size distribution, particle concentration, and zeta potential of PP-L-EVLPs. To evaluate the membrane integrity of PP-L-EVLPs, a Triton X-100 lysis assay was performed. Specifically, purified extracellular vesicle-like particles derived from *P. polyphylla* var. *yunnanensis* leaves were diluted to an appropriate concentration and maintained on ice. Subsequently, 5 μl of 10%, 5%, 1%, or 0.25% Triton X-100 solution was added to 45 μl of PP-L-EVLPs. The mixtures were vortexed thoroughly and incubated on ice for 30 min, with additional vortexing at 10-min intervals. Finally, the samples were analyzed using nano-flow cytometry (nFCM).

### Primary culture of PDLSCs

PDLSCs were isolated from healthy premolars extracted from orthodontic patients (17 to 22 years) under ethical approval (KYKQ2024MEC0154, Kunming Medical University Affiliated Stomatological Hospital). Teeth were rinsed in cold PBS containing antibiotics, and periodontal ligament tissues were scraped from the root middle third. Tissue fragments were centrifuged (1,000*g*, 2 min) and cultured in Dulbecco’s modified Eagle’s medium (DMEM)/F12 with 20% fetal bovine serum (FBS). After 2 to 3 h of adherence in a 37 °C/5% CO₂ incubator, flasks were horizontally incubated, with medium refreshed every 3 d. At 80% confluence, cells were passaged (0.25% trypsin) at a 1:3 ratio and maintained in 10% FBS DMEM/F12.

### Multidirectional differentiation potential of PDLSCs

#### Colony formation

Third-passage (P3) PDLSCs (200 cells per dish) were cultured for 14 d, fixed (4% paraformaldehyde), and stained with crystal violet. Colonies were imaged under phase-contrast microscopy.

#### Osteogenesis

PDLSCs (1 × 10^5^ cells per well) were induced with osteogenic medium (50 μM ascorbate, 10 mM β-glycerophosphate, 100 nM dexamethasone) for 21 d. Mineralized nodules were stained with Alizarin Red.

#### Adipogenesis

Overconfluent PDLSCs were induced with adipogenic medium (0.5 mM 3-isobutyl-1-methylxanthine, 1 μM dexamethasone, 10 μg/ml insulin) for 28 d. Lipid droplets were stained with Oil Red O.

### Surface marker analysis

PDLSCs were trypsinized, washed, and incubated with antibodies [CD29-phycoerythrin (PE), CD44-PE, CD90-PE, CD105-PE, CD34-fluorescein isothiocyanate (FITC), CD45-FITC] for 30 min at 4 °C. After washing, cells were analyzed by flow cytometry (NOVO CYTE D2040R, Agilent Technologies).

### Cell viability assay

P3 PDLSCs at 80% confluence were trypsinized, counted, and seeded into 96-well plates at a density of 3,000 cells per well. After cell attachment, the culture medium was replaced with serum-free DMEM/F12 for overnight serum starvation. The following day, the medium was removed, and cells were incubated for 24, 48, or 72 h with PP-L-EVLPs at concentrations of 50, 100, 200, 400, 800, 1,600, and 3,200 ng/ml (8 groups, including a control group). After incubation, the old medium was discarded, and 90 μl of fresh medium supplemented with 10 μl of cell counting kit-8 (CCK-8) solution was added to each well. Cells were incubated at 37 °C for 2 h in the dark. Absorbance (optical density values) at 490 nm was measured using a microplate reader (VICTOR Nivo 3S, Revvity).

### Comprehensive cell safety evaluation

To assess biocompatibility and cytotoxicity, PDLSCs were treated with varying concentrations of PP-L-EVLPs (50 to 800 ng/ml) for 24 and 48 h. Cell cycle (Beyotime, China) and apoptosis [Annexin V-FITC/propidium iodide (PI) staining; Proteintech, USA] were analyzed by flow cytometry (NOVO CYTE D2040R, Agilent Technologies, Santa Clara, CA).

### Cell uptake of PP-L-EVLPs

P3 PDLSCs at 80% confluence were trypsinized, counted, and seeded into 6-well plates at a density of 1 × 10^5^ cells per well. After cell attachment, the culture medium was replaced with serum-free DMEM/F12 for overnight starvation. The following day, cells were treated with PP-L-EVLPs at concentrations of 50, 100, and 200 ng/ml (diluted in fresh medium) and incubated for 24, 48, or 72 h under standard culture conditions (37 °C, 5% CO₂). For fluorescent labeling of PP-L-EVLPs, particles were incubated with 5 μM Dil (Beyotime, China) for 20 min at 37 °C, followed by centrifugation (12,000*g*, 20 min) to remove unbound dye. Labeled PP-L-EVLPs were resuspended in PBS and applied to PDLSCs as described. After treatment, cells were processed for analysis using a NovoCyte D2040R flow cytometer (Agilent Technologies, Santa Clara, CA) and imaged under a laser scanning confocal microscope (A1, Nikon, Japan).

### Cell migration assay

Under sterile conditions in a laminar flow hood, a Culture-Insert (ibidi GmbH, Germany) was aseptically placed at the center of a 24-well plate using sterile microforceps. P3 cells were seeded into the Culture-Insert and incubated overnight at 37 °C with 5% CO₂. After confirming confluent cell growth within the Culture-Insert region under a phase-contrast microscope, the insert was gently removed with forceps to generate a uniform 500-μm-wide scratch. Cells were washed 3 times with PBS to remove debris and dead cells, followed by treatment according to experimental groups. Images of the scratch region were captured at 0, 12, 24, 36, and 48 h using an inverted phase-contrast microscope (Olympus CKX53, Japan). The wound area was quantified using ImageJ software by measuring the scratch closure rate relative to the initial wound area.

### Effect of PP-L-EVLPs on osteogenic differentiation of PDLSCs

PDLSCs at 80% confluence were cultured in osteogenic induction medium containing PP-L-EVLPs at concentrations of 0, 50, 100, or 200 ng/ml. Cells cultured in osteogenic medium (OM) without PP-L-EVLPs served as the blank group. The medium was refreshed every 3 d. After 3 d of differentiation, alkaline phosphatase (ALP) activity was quantified using the ALP staining solution (Beyotime, China). Mineralized nodule formation was assessed by Alizarin Red S staining (Sigma-Aldrich, USA) after 7 d. Osteogenic marker genes (*ALP*, *RUNX2*, and *OPN*) were analyzed via reverse transcription quantitative polymerase chain reaction (RT-qPCR) using SYBR Green Master Mix (Vazyme, China), with β-actin as the internal reference. The primer sequences used are listed in Table S1.

Protein expression of osteogenic markers (ALP, RUNX2, and OPN) was evaluated by Abby Simple Western System (ProteinSimple, USA). Compass software (ProteinSimple) was used to acquire the data and then perform image reconstruction and examine chemiluminescence signal intensity. The following primary antibodies were used: anti-ALP (catalog no. PA5-106391, 1:100, Invitrogen), anti-OPN (catalog no. 80912-4-RR, 1:200, Proteintech), anti-RUNX2 (catalog no. 82636-2-RR, 1:2,000, Proteintech), and anti-β-actin (catalog no. ab6276, 1:10,000, Abcam). All experiments were performed in triplicate.

### ELISA was performed to detect the expression levels of inflammatory cytokines

PDLSCs were seeded in 6-well plates at a density of 1 × 10^5^ cells per well. After adherence, the medium was replaced with serum-free medium for 12-h synchronization. The experimental groups were stimulated with 1 μg/ml lipopolysaccharide (LPS) from *Porphyromonas gingivalis* (InvivoGen, USA) for 24/48 h, while control groups were treated with serum-free medium alone. Cell supernatants were collected and centrifuged at 1,200*g* for 10 min to remove debris. Human interleukin-6 (IL-6)/IL-8 enzyme-linked immunosorbent assay (ELISA) kits (HUABIO, China) were used strictly according to the manufacturer’s instructions. Absorbance was measured using a microplate reader (VICTOR Nivo 3S, Revvity).

### Experimental animals

All experimental protocols were approved by the Animal Ethics Committee of Kunming Medical University (approval no.: KMMUX202412015) and complied with ARRIVE guidelines. Male SD rats (6 to 8 weeks old, body weight ≈ 220 g) were obtained from the Experimental Animal Center of Kunming Medical University [license no.: SCXK (Yunnan) K2020-0004] and maintained under specific pathogen-free (SPF) conditions (temperature: 22 ± 1 °C, humidity: 55 ± 5%, 12-h light/dark cycle) with ad libitum access to sterilized food and water.

Rats were anesthetized via intraperitoneal injection of pentobarbital sodium (40 mg/kg). A standardized critical-sized defect (3 mm × 2 mm × 1 mm) was created in the mandibular alveolar bone using a dental trephine bur under aseptic conditions.

### Micro-CT analysis

At 4, 6, and 8 weeks post-surgery, alveolar bone samples were harvested, fixed in 4% paraformaldehyde, and scanned using a high-resolution micro-computed tomography (CT) system [NEMO NMC-100, PINGSENG Healthcare (Kunshan) Inc., China] at 90 kV, 60 μA. Bone volume (BV), bone surface (BS), bone volume fraction (BV/TV, %), bone surface/tissue volume (BS/TV, %), trabecular thickness (Tb.Th, mm), trabecular number (Tb.N), trabecular separation (Tb.Sp, mm), bone mineral density (BMD), and bone mineral content (BMC) were calculated using Avatar 3.0 software. Representative 3-dimensional (3D) reconstructions were generated with Avatar 3.0 software.

### Histological staining

Mandibular bone samples were harvested at 2, 4, and 6 weeks post-surgery (*n* = 6 per group), fixed in 4% paraformaldehyde (Biosharp, China) for 24 h, and decalcified in 10% EDTA (pH 7.4, Sigma-Aldrich, USA) for 4 weeks. Tissues were dehydrated in graded ethanol, embedded in paraffin, and sectioned into 5-μm slices using a rotary microtome (Leica RM2235, Germany). Hematoxylin and eosin (H&E) staining and Masson’s trichrome staining (Servicebio, China) were performed to evaluate osteogenesis and collagen deposition, respectively. Slides were scanned (KF-PRO-005, KFBIO, China) for ALP, OPN, BSP, RUNX2, PERIOSTIN, and FIBRONECTIN using K-viewer 1.7.0.27. Quantitative analysis of newly formed bone area (percentage) was performed using the HALO image analysis platform (Indica Labs, USA), with 3 randomly selected fields assessed per sample.

### In vivo safety evaluation

Heart, liver, spleen, lung, kidney, and pancreatic tissues were harvested at 2, 4, and 6 weeks post-surgery. Tissues were fixed in 4% paraformaldehyde (Biosharp, China) for 24 h, rinsed under running water for 24 h, dehydrated through a graded ethanol series, embedded in paraffin, and sectioned into 5-μm slices. H&E staining and γ-H2A.X (1:1,000 dilution, Abcam, UK) immunohistochemical staining were performed to assess histopathological changes and DNA damage, respectively. Slides were scanned (KF-PRO-005, KFBIO, China), and image acquisition was performed using K-viewer 1.7.0.27.

### Statistical analysis

Data were analyzed using SPSS 19.0 statistical software. Experimental results are expressed as mean ± standard deviation. Comparisons between 2 groups were performed using an independent samples *t* test. For comparisons involving 3 or more groups, one-way analysis of variance (ANOVA) was applied, with the significance level set at a 2-tailed α = 0.05.

## Results

### Physicochemical characterization of PP-L-EVLPs

**Fig. 1.**
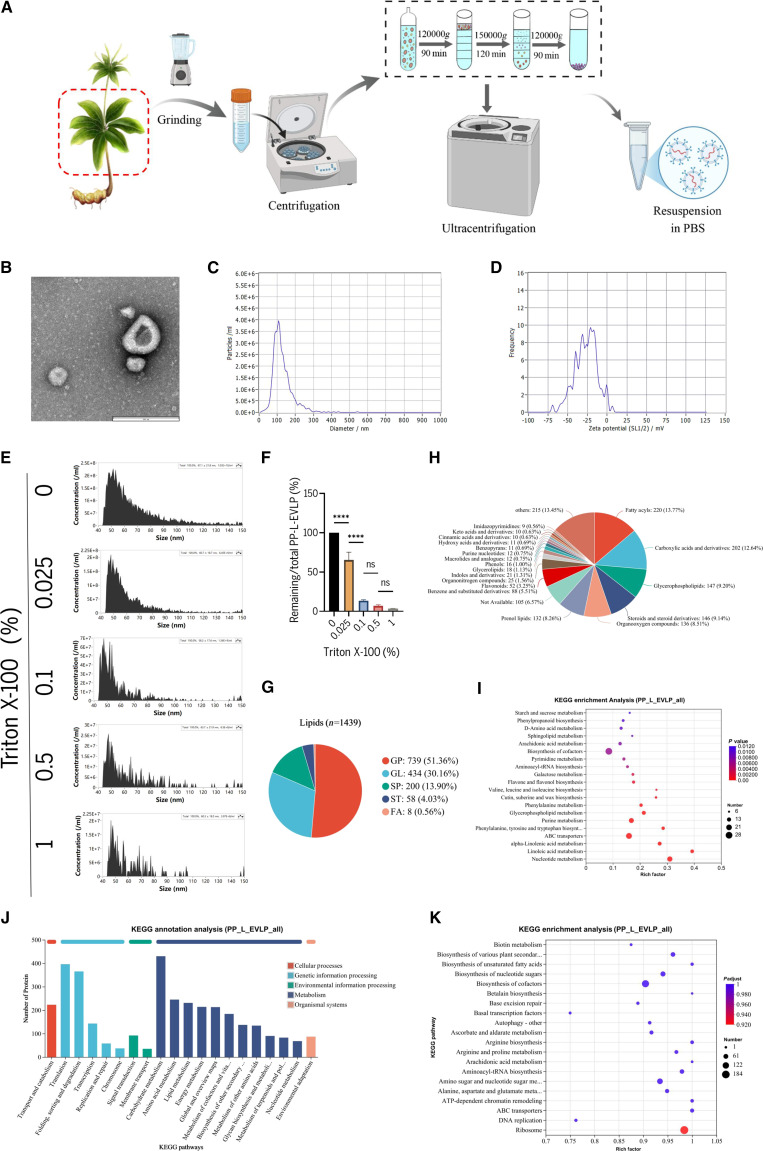
Identification and analysis of PP-L-EVLPs. (A) Schematic diagram illustrating the process of isolating and purifying PP-L-EVLPs. (B) Morphology of PP-L-EVLPs determined by TEM (scale bar, 200 nm). (C) Particle size distribution of PP-L-EVLPs. (D) Surface charge of PP-L-EVLPs. (E and F) PP-L-EVLPs were incubated with Triton X-100, and its purity is indirectly demonstrated by membrane rupture efficiency (*n* = 3). ****P < 0.0001. (G) Identification of PP-L-EVLPs components by lipidomics. (H and I) Untargeted metabolomics profiling and KEGG enrichment analysis of PP-L-EVLPs components. (J and K) Identification and KEGG pathway analysis of PP-L-EVLPs protein components via proteomics.

Extracellular vesicles derived from *P. polyphylla* var. *yunnanensis* leaves were isolated via sequential ultracentrifugation followed by sucrose density gradient centrifugation (Fig. 1A). TEM revealed that PP-L-EVLPs displayed the characteristic cup-shaped morphology with a bilayer membrane structure (Fig. 1B). NTA confirmed a size distribution of 50 to 150 nm, with an average diameter of 104.8 ± 3.7 nm (Fig. 1C). Zeta potential measurements demonstrated a surface charge of −27.03 ± 0.86 mV, consistent with the negatively charged surface typical of extracellular vesicles (Fig. 1D). The purity of PP-L-EVLPs was assessed using Triton X-100 membrane lysis assays. A significant reduction in intact vesicles was observed with increasing Triton X-100 concentrations (0 to 1% v/v), stabilizing at ≥0.1% with a purity of 96.68% (Fig. 1E and F), meeting the stringent requirements for subsequent functional experiments. Lipidomic profiling identified 1,439 lipid species, including 739 glycerophospholipids (GPs; 51.36%), 434 glycerolipids (GLs; 30.16%), 200 sphingolipids (SPs; 13.90%), 58 sterol lipids (STs; 4.03%), and 8 fatty acyls (FAs; 0.56%) (Fig. 1G). Untargeted metabolomic analysis revealed predominant metabolites: FAs (13.77%), carboxylic acids and derivatives (12.64%), GPs (9.20%), steroids and steroid derivatives (9.14%), organooxygen compounds (8.51%), and prenol lipids (8.26%), which were implicated in regulating diverse biosynthetic and metabolic pathways (Fig. 1H and I). Proteomic profiling demonstrated that PP-L-EVLPs proteins participate in genetic information processing, environmental information processing, cellular processes, metabolism, and organismal systems (Fig. 1J and K). Collectively, PP-L-EVLPs contain mammalian exosome-like components that coordinately regulate organismal development and physiological processes.

### Primary culture and characterization of PDLSCs

PDLSCs were isolated and expanded using the tissue explant adherence method, with subculturing performed at approximately 80% confluence. The PDLSCs exhibited a spindle-shaped morphology, demonstrated robust clonogenic potential, and possessed osteogenic and adipogenic differentiation capacities (Fig. S1A to D). Flow cytometric analysis of surface markers revealed negative expression of hematopoietic lineage markers CD34 (0.58%) and CD45 (0.26%), while mesenchymal stem cell markers CD29 (99.37%), CD44 (99.85%), CD90 (99.03%), and CD105 (99.89%) were strongly expressed (Fig. S1E).

### Effects of PP-L-EVLPs on the biological characteristics of PDLSCs

**Fig. 2.**
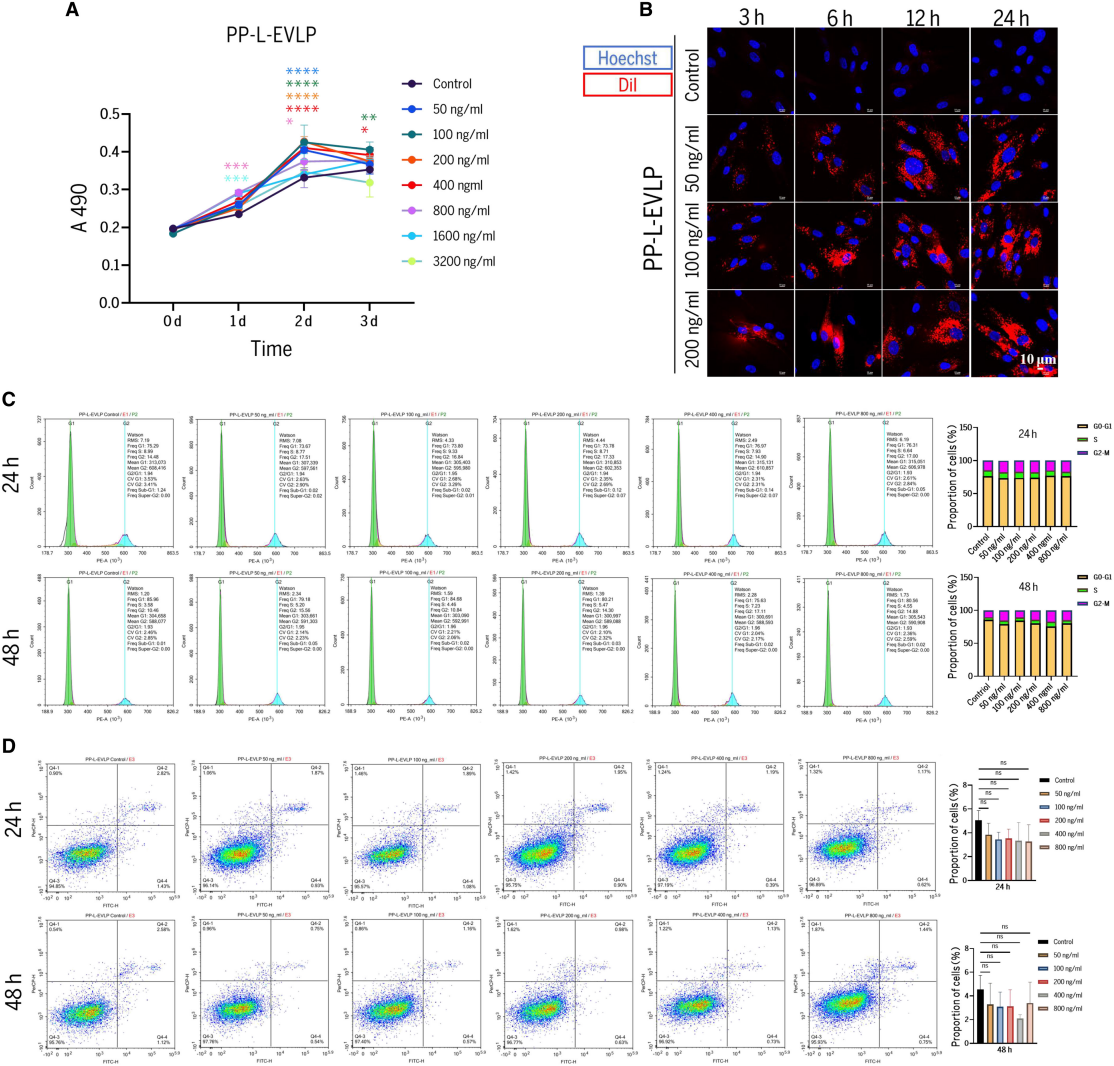
The effects of PP-L-EVLPs on the biological functions of PDLSCs. (A) CCK-8 assay evaluating PP-L-EVLPs on PDLSC proliferation (*n* = 3). (B) Fluorescence microscopy images of PDLSCs were treated with Dil-labeled PP-L-EVLPs (50, 100, and 200 ng/ml) for 3, 6, 12, and 24 h (scale bar, 10 μm). (C) Flow cytometry-based evaluation of PP-L-EVLPs-induced cell cycle changes in PDLSCs with quantitative analysis (*n* = 3). (D) Flow cytometry assessing the effects of PP-L-EVLPs on PDLSC apoptosis levels and quantitative analysis (*n* = 3). *P < 0.05, **P < 0.01.

CCK-8 assays revealed dose-dependent effects of PP-L-EVLPs on the proliferation of PDLSCs over 0 to 3 d (Fig. 2A). Compared to the control group, low concentrations of PP-L-EVLPs promoted PDLSC proliferation starting at day 1. However, concentrations ≥1,600 ng/ml suppressed cell growth, particularly on day 3.

Laser scanning confocal microscopy demonstrated efficient uptake of PP-L-EVLPs by PDLSCs as early as 3 h post-treatment (Fig. 2B). Notably, 50, 100, and 200 ng/ml PP-L-EVLPs treatments resulted in progressive accumulation of PP-L-EVLPs around PDLSCs over time (Fig. 2B and Fig. S2).

Flow cytometric analysis of cell cycle progression and apoptosis showed no statistically significant differences in PDLSCs treated with 50 to 800 ng/ml PP-L-EVLPs for 24 or 48 h compared to controls, indicating the safe applicability of PP-L-EVLPs at concentrations ≤800 ng/ml (Fig. 2C and D).

### The effects of PP-L-EVLPs on the migration and osteogenic differentiation of PDLSCs

**Fig. 3.**
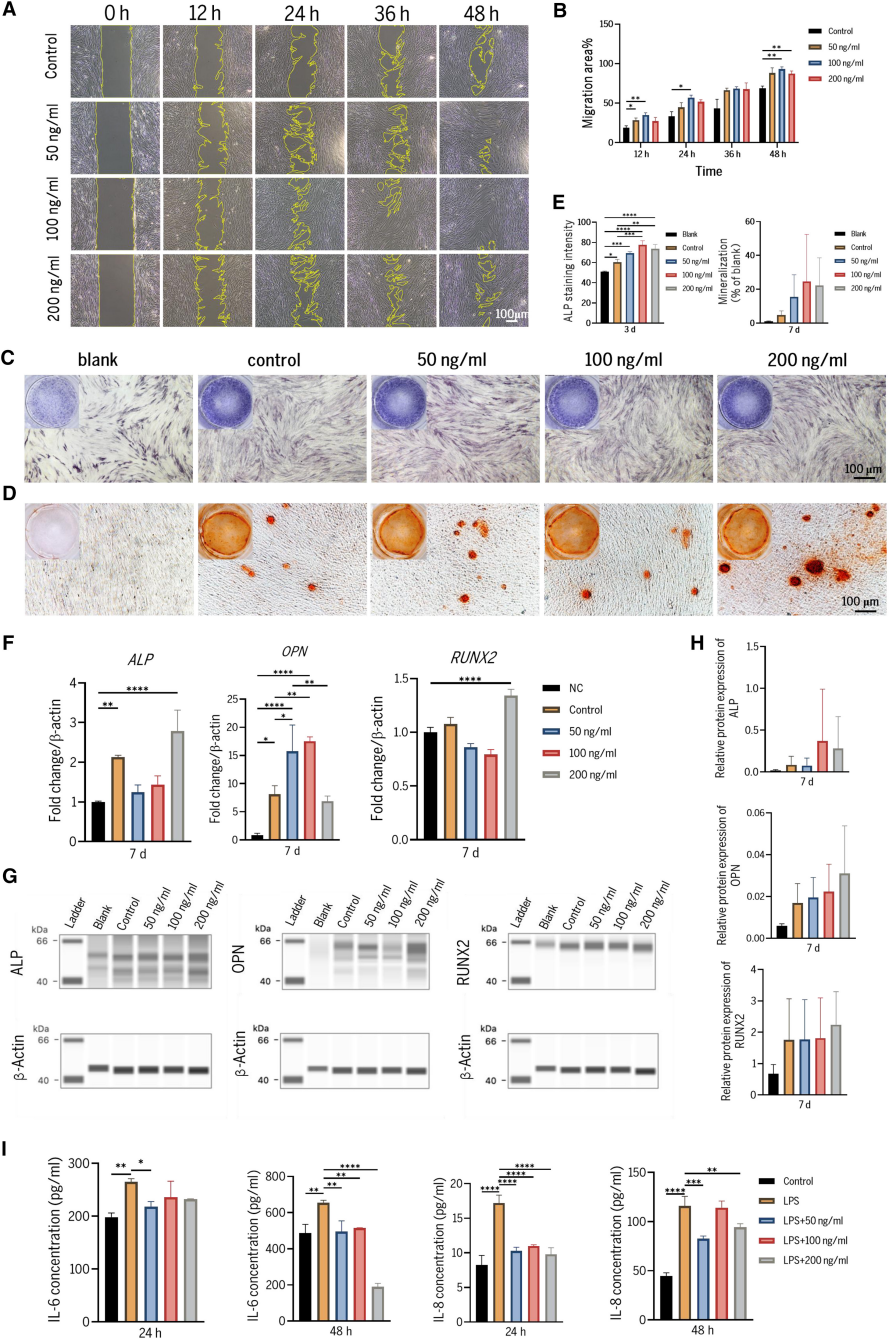
PP-L-EVLPs promote migration and osteogenic differentiation of PDLSCs in vitro. (A) Scratch assay to assess the effects of PP-L-EVLPs on PDLSC migration. (B) Quantitative analysis of migration rates (*n* = 3). (C) Alkaline phosphatase (ALP) staining images after 3 d of PP-L-EVLPs induction (scale bar, 100 μm). (D) Alizarin Red S staining (ARS) images of PDLSCs on day 14 osteogenic differentiation (scale bar, 100 μm). (E) Quantitative analysis of ALP and Alizarin Red S staining (*n* = 3). (F) Effect of PP-L-EVLPs on osteogenesis-related marker mRNA levels in PDLSCs on day 7 of osteogenesis differentiation was detected by PCR. Data are presented as mean ±SD (*n* = 3). (G) Effect of PP-L-EVLPs on osteogenesis-related marker protein levels in PDLSCs on day 7 of osteogenesis differentiation was detected by Western blot. (H) Quantitative analysis of protein expression levels. (I) ELISA was performed to detect the expression levels of inflammatory cytokines. Data are presented as mean ±SD (*n* = 3). *P < 0.05, **P < 0.01, ***P < 0.001, ****P < 0.0001.

Scratch assays further revealed that PP-L-EVLPs significantly enhanced PDLSC migration (Fig. 3A and B). At concentrations of 50, 100, and 200 ng/ml, PP-L-EVLPs-treated groups exhibited markedly higher migration rates than controls at all time points (12, 24, 36, and 48 h), confirming its potent pro-migratory effects.

Osteogenic differentiation of PDLSCs was assessed through ALP staining at day 3, quantification of osteogenic-related genes and proteins at day 7, and Alizarin Red S staining at day 14 (Fig. 3 and Fig. S3). Compared with the control group, PP-L-EVLPs-treated PDLSCs exhibited increased deposition of bluish purple precipitates on cell membranes in ALP staining (Fig. 3C), with quantitative analysis confirming enhanced deposition at day 3 (Fig. 3E), indicating potentiated early osteogenic potential. At day 14, Alizarin Red S staining with quantitative analysis revealed sustained pro-osteogenic effects, demonstrating increased mineralized nodule formation in PP-L-EVLPs-treated groups compared to controls (Fig. 3D and E). qPCR analysis of osteogenic markers at day 7 revealed significant up-regulation of key genes, including *ALP*, *OPN*, and *RUNX2* across all tested PP-L-EVLPs concentrations relative to untreated cells (Fig. 3F). Western blot analysis also showed the same trend (Fig. 3G and H). PP-L-EVLPs significantly suppressed the protein expression levels of inflammatory cytokines IL-6 and IL-8 in *Porphyromonas gingivalis*-LPS-induced PDLSCs (Fig. 3I).

### Radiographic evaluation of PP-L-EVLPs in periodontal defect repair

**Fig. 4.**
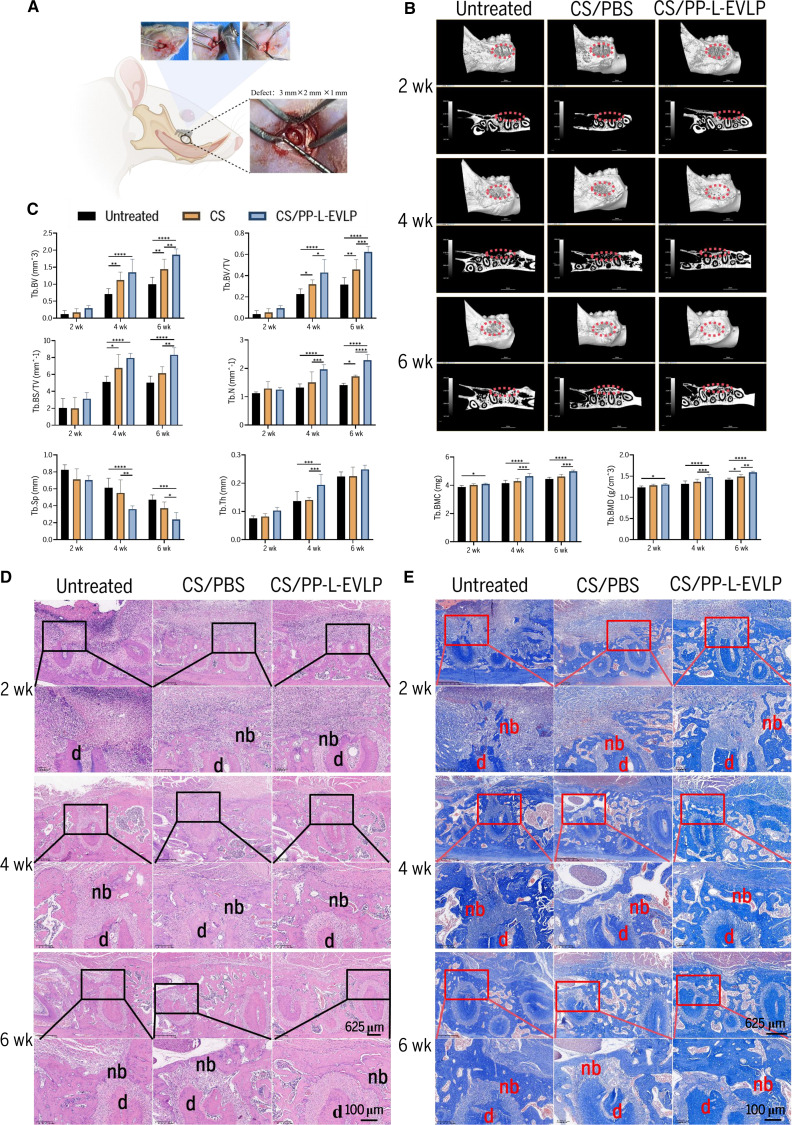
PP-L-EVLPs promoted periodontal tissue regeneration in rats. (A) Establishment of a periodontal defect model in SD rats. (B) Representative micro-CT reconstruction images showing new bone formation in different groups at 2, 4, and 6 weeks. The dashed box delineates the periodontal defect area. (C) Quantitative assessment of bone volume (BV), bone volume fraction (BV/TV, %), bone surface/tissue volume (BS/TV, %), trabecular thickness (Tb.Th, mm), trabecular number (Tb.N), trabecular separation (Tb.Sp, mm), bone mineral density (BMD), and bone mineral content (BMC) (*n* = 6). (D) Assessment of periodontal tissue repair in different groups via H&E staining after 2, 4, and 6 weeks of treatment. High scale bar, 100 μm; low scale bar, 625 μm. (E) Assessment of periodontal tissue repair in different groups via Masson’s trichrome staining after 2, 4, and 6 weeks of treatment. High scale bar, 100 μm; low scale bar, 625 μm. *P < 0.05, **P < 0.01, ***P < 0.001, ****P < 0.0001.

To assess the therapeutic efficacy of PP-L-EVLPs in periodontal tissue repair, a periodontal defect model was established in SD rats (Fig. 4A). Collagen sponges loaded with PP-L-EVLPs were implanted into the defect sites. Micro-CT scanning and 3D reconstruction were performed on mandibular specimens from SD rats with periodontal defects, including untreated controls, collagen sponge (CS/PBS)-treated groups, and collagen sponge-loaded PP-L-EVLPs (CS/PP-L-EVLPs) groups (Fig. 4B). At 2 weeks post-surgery, no significant new bone formation was observed in any group. By 4 weeks, all groups exhibited nascent bone regeneration, with the CS/PP-L-EVLPs group demonstrating accelerated defect closure compared to controls. At 6 weeks, progressive healing was evident across all groups, with the CS/PP-L-EVLPs group showing denser new bone formation and superior repair outcomes (Fig. 4B). Quantitative analysis of bone morphometric parameters revealed significant enhancements in the CS/PP-L-EVLPs group at 4 weeks: BV, BS, BV/TV, BS/TV, Tb.Th, Tb.N, BMD, and BMC were markedly elevated compared to untreated controls (*P* < 0.01), while Tb.Sp was significantly reduced (*P* < 0.01). Similar trends persisted at 6 weeks (Fig. 4C). These findings conclusively demonstrate that PP-L-EVLPs promote alveolar bone regeneration and enhance structural restoration in rat periodontal defects.

### Histological assessment of PP-L-EVLPs in alveolar bone defect repair

Following specimen fixation, paraffin-embedded sections were prepared for H&E staining (Fig. 4D) and Masson’s trichrome staining (Fig. 4E). H&E staining at 2 weeks revealed abundant connective tissue formation in both the CS/PBS and CS/PP-L-EVLPs groups. Over time, the CS/PP-L-EVLPs group exhibited markedly enhanced new bone formation with a denser trabecular structure compared to other groups, particularly at 4 weeks. Masson’s trichrome staining corroborated this trend, demonstrating progressive collagen organization and matrix maturation in the CS/PP-L-EVLPs group. Collectively, these histological findings confirm that PP-L-EVLPs significantly enhance the structural and compositional restoration of periodontal defects in SD rats.

### Immunohistochemistry evaluation of PP-L-EVLPs in bone defect repair

**Fig. 5.**
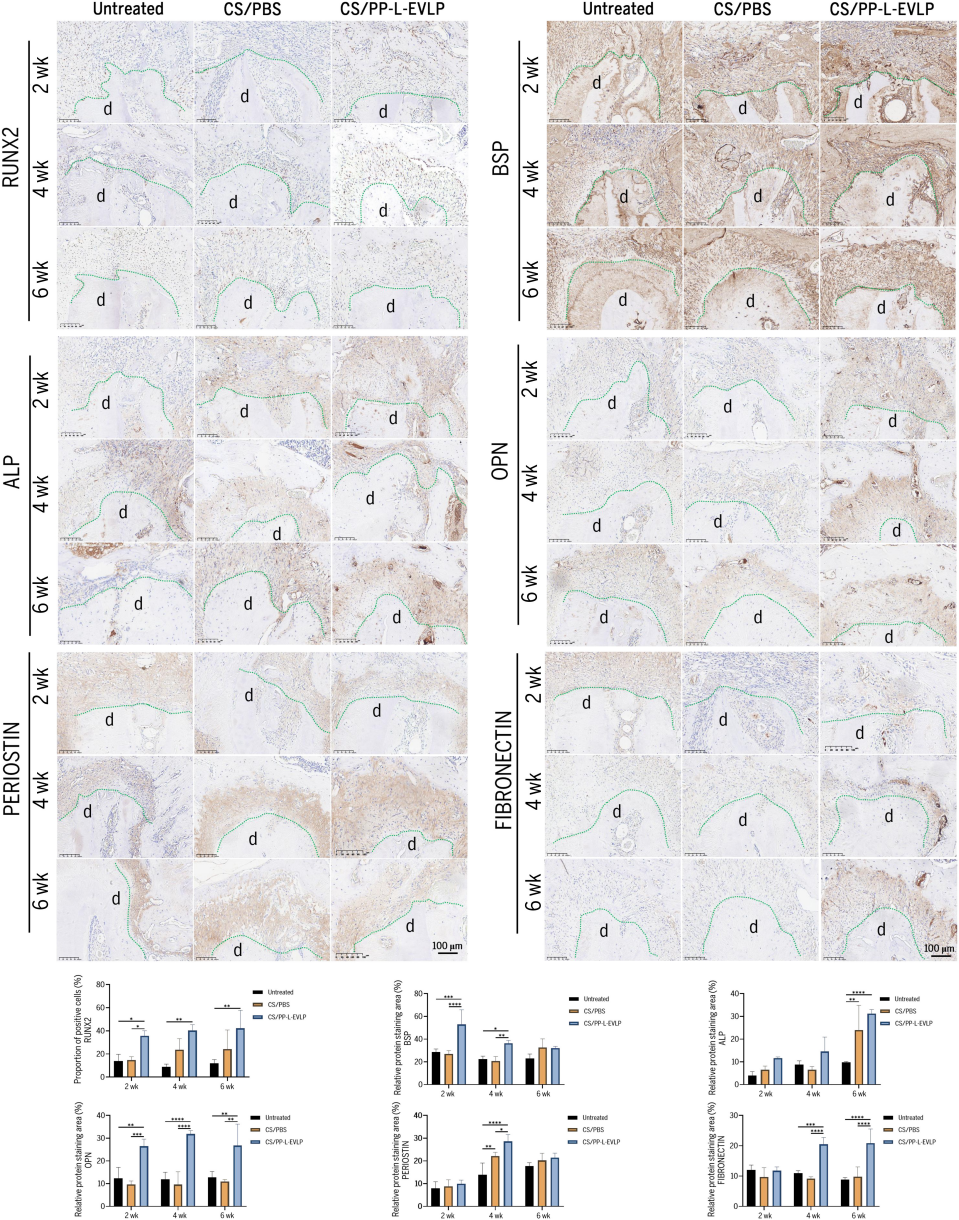
Immunohistochemical analysis of osteogenic and periodontal markers in rat periodontal defect tissues in vivo (scale bar, 100 μm). d, dentin. *P < 0.05, **P < 0.01, ***P < 0.001, ****P < 0.0001.

At 2 and 4 weeks post-implantation, a significant increase in RUNX2-positive nuclei was observed around the defective tooth roots in the PP-L-EVLPs group compared to the untreated and PBS groups, indicating enhanced early osteogenic commitment (Fig. 5). Representative immunohistochemical staining images of ALP, OPN, BSP, PERIOSTIN, and FIBRONECTIN further demonstrated that the PP-L-EVLPs group exhibited deeper and more extensive staining than the control groups. Quantitative analysis using HALO image analysis software confirmed that the expression levels of all 6 markers were significantly up-regulated in the PP-L-EVLPs group (Fig. 5). These findings collectively suggest that PP-L-EVLPs markedly promote osteogenic differentiation and periodontal tissue regeneration.

### In vivo safety evaluation of PP-L-EVLPs

**Fig. 6.**
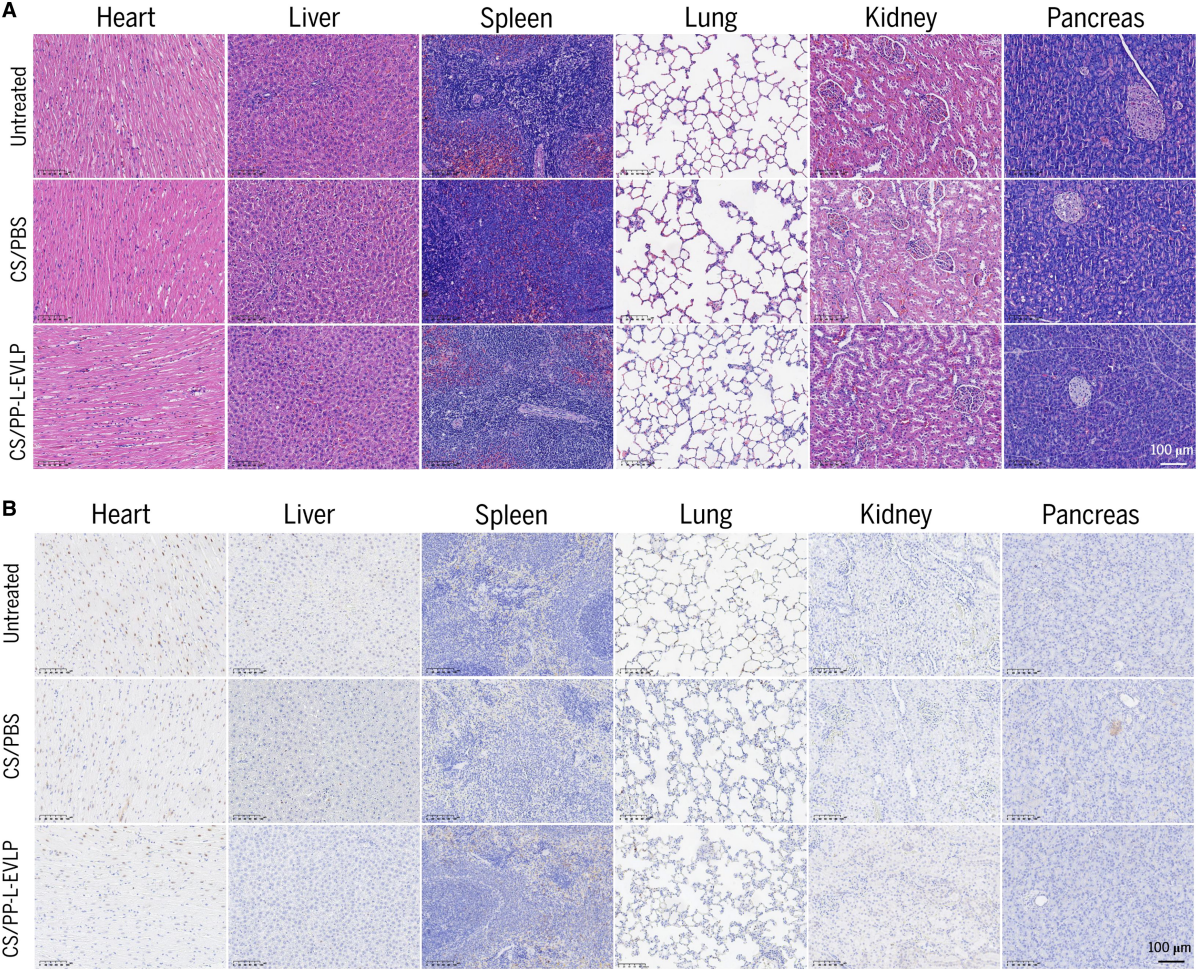
Safety evaluation of PP-L-EVLPs in vivo. (A) H&E staining of different organs showed no obvious histological changes after 6 weeks of treatment. Scale bar, 100 μm. (B) Immunohistochemistry staining was used to observe the expression of γ-H2A.X. Scale bar, 100 μm.

Heart, liver, spleen, lungs, kidneys, and pancreas were harvested at 2, 4, and 6 weeks post-treatment, fixed in paraformaldehyde, and subjected to H&E staining and γ-H2A.X immunohistochemical analysis. Histopathological evaluation revealed no evidence of inflammatory infiltration, necrosis, or fibrotic lesions in PP-L-EVLPs-treated rats across all time points compared to controls (Fig. 6A and Fig. S4A). Tissue architecture in the heart, liver, spleen, lungs, kidneys, and pancreas remained indistinguishable from that of the control group. Immunohistochemical quantification of γ-H2A.X-positive cells showed no statistically significant differences between PP-L-EVLPs-treated and control groups in any examined organs, indicating negligible genotoxic risk (Fig. 6B and Fig. S4B). These findings collectively demonstrate the biosafety of PP-L-EVLPs for in vivo applications in SD rats.

## Discussion

The pathogenesis of periodontitis involves chronic inflammatory infiltration that disrupts periodontal immune homeostasis, leading to a reduction in PDLSC reserves, loss of soft tissue attachment, and progressive alveolar bone resorption [3,20]. Regeneration of periodontal tissues remains a significant challenge in periodontal therapy due to the complex spatial architecture required for functional reconstruction, including cementum, directionally aligned periodontal ligament fibers, and alveolar bone [21]. In this study, extracellular vesicles derived from PP-L-EVLPs, a traditional medicinal herb native to Yunnan, were systematically investigated for the first time to evaluate their regulatory effects on the biological behavior and osteogenic differentiation of PDLSCs. Additionally, their regenerative efficacy and biosafety were validated using an alveolar bone defect model in SD rats.

Chinese herbal medicine-derived extracellular vesicles (CHMEVs) are natural nanoscale carriers enriched with bioactive components (e.g., small-molecule metabolites, nucleic acids, and functional proteins) from their parent plants, exhibiting multi-component synergistic effects and high biocompatibility [22]. Compared to traditional herbal extracts, CHMEVs significantly enhance the bioavailability of active ingredients and enable targeted delivery via membrane fusion or endocytosis, demonstrating unique advantages in tissue repair [23,24]. In this study, PP-L-EVLPs with a characteristic cup-shaped bilayer membrane structure was successfully isolated via ultracentrifugation combined with sucrose density gradient centrifugation, achieving a purity of 96.68%. The observed discrepancy in PP-L-EVLPs particle size measurements between TEM and NTA is likely attributable to particle deformation or aggregation during TEM specimen preparation, as established in extracellular vesicle characterization methodologies [25]. Multi-omics analyses (proteomics, lipidomics, and untargeted metabolomics) confirmed that PP-L-EVLPs carry 739 functional proteins (e.g., cytoskeleton-regulating proteins), 1,439 lipid species (51.36% GPs), and diverse terpenoid/alkaloid metabolites, suggesting cross-species regulatory potential akin to mammalian exosomes and broad regulatory capacity in tissue regeneration. Our results demonstrated that PP-L-EVLPs are enriched in multiple classes of bioactive molecules, including flavonoids, GPs, SPs, and arachidonic acid metabolites, which have been previously reported to be involved in osteogenic regulation and bone tissue remodeling [9]. Moreover, Kyoto Encyclopedia of Genes and Genomes (KEGG) enrichment analysis of the differentially abundant proteins and metabolites revealed significant enrichment in several osteogenesis-related signaling pathways, such as flavone and flavonol biosynthesis, arachidonic acid metabolism, SP metabolism, and GP metabolism, suggesting a potential mechanistic basis for their pro-osteogenic effects.

Extracellular vesicles regulate recipient cell proliferation, migration, differentiation, and anti-inflammation by delivering bioactive substances [26,27]. The CCK-8 assay found that low concentrations of PP-L-EVLPs (50 to 800 ng/ml) significantly promoted PDLSC proliferation, whereas high concentrations (≥1,600 ng/ml) exhibited inhibitory effects. Notably, treatment of PDLSCs with PP-L-EVLPs at concentrations up to 800 ng/ml for 24 or 48 h induced no signs of cell cycle arrest (Fig. 2C), apoptosis (Fig. 2D), or significant increases in cell death (Fig. S5), confirming its safety for in vitro applications. These results align with recent studies: *Aloe vera* peel-derived EVs (A-EVs) demonstrated low cytotoxicity in HaCaT cells [28], and *Morinda officinalis*-derived EVs (MOEVs) promoted osteoblast proliferation via mitogen-activated protein kinase (MAPK) pathway activation [29].

Further experiments confirmed the osteoinductive effects of PP-L-EVLPs. At day 3 post-osteogenic induction, significantly intensified bluish purple precipitates were observed in PP-L-EVLPs-treated groups via ALP staining, with quantification confirming enhanced deposition. After 7 d of treatment, key osteogenic genes (*ALP*, *RUNX2*, and *OPN*) were up-regulated at both mRNA and protein levels. By day 14, Alizarin Red S staining with quantitative analysis demonstrated increased mineralized nodule formation in PP-L-EVLPs-treated groups compared to controls. The data demonstrated significant pro-osteogenic effects on PDLSCs throughout the 50 to 800 ng/ml spectrum (Fig. S3A to D). However, given the documented moderate toxicity of *P. polyphylla* var. *yunnanensis* in the Chinese Pharmacopoeia (2020 Edition) and our biosafety validation data, we prioritized the 50 to 200 ng/ml concentration window for subsequent experiments. These findings align with the shared mechanisms of plant-derived vesicle-mediated osteogenesis: Plum-derived exosome-like nanovesicles induce osteoblast differentiation and reduce osteoclast activation [30], while apple-derived nanovesicles up-regulate *BMP-2/4* and *RUNX2* by activating the MAPK/ERK (extracellular signal-regulated kinase) pathway [31].

In vivo experiments further substantiated PP-L-EVLP’s therapeutic potential. A standardized periodontal defect (3 mm × 2 mm × 1 mm) was surgically created at the buccal mesial site of the right mandibular first molar in SD rats. Our research group has successfully established and validated this model in prior studies, confirming its reliability and reproducibility for evaluating periodontal tissue regeneration [32–34]. In the SD rat alveolar bone defect model, PP-L-EVLPs exhibited excellent biosafety: No inflammatory infiltration, fibrosis, or γ-H2A.X-positive cell accumulation was observed in major organs (heart, liver, spleen, etc.) at 2 to 6 weeks post-surgery, consistent with prior CHMEV safety reports [22]. Micro-CT analysis revealed significant bone regeneration in the PP-L-EVLPs-treated group at 4 weeks, with elevated BV, BS, BV/TV, BS/TV, Tb.Th, Tb.N, BMD, and BMC compared to controls. Histological evaluations (H&E and Masson staining) further confirmed its ability to promote organized collagen deposition and bone matrix maturation. Beyond these structural outcomes, we investigated the immunomodulatory mechanism underlying the enhanced regeneration. A comprehensive time-course analysis of macrophage polarization revealed a compelling shift in the immune response (Fig. S6). PP-L-EVLPs treatment promoted a significant transition from pro-inflammatory iNOS^+^ M1 macrophages toward pro-healing CD163^+^ M2 macrophages at the defect site during the regenerative phases (4 to 6 weeks). This active immunomodulation, fostering a tissue-reparative microenvironment, likely constitutes a key mechanism through which PP-L-EVLPs enhance bone and periodontal tissue regeneration. The regenerative prowess of PP-L-EVLPs aligns with and expands the known mechanisms of CHMEVs. For instance, *Rhizoma Drynariae-derived* nanovesicles (RDNVs) enhance osteogenic differentiation of human bone marrow mesenchymal stem cells (hBMSCs) via estrogen receptor α (ERα) targeting [35]; *Pueraria lobata*-derived EVs reverse osteoporosis by modulating hBMSC mineralization [36]; and *Zingiber officinale*-derived EVs (Gr-EVs) synergistically reduce bone resorption by inhibiting *P. gingivalis* virulence [37]. Furthermore, PP-L-EVLPs exhibited excellent systemic biosafety, with no signs of inflammatory infiltration or toxicity in major organs, consistent with prior safety profiles of CHMEVs [22].

As the first systematic study of *P. polyphylla*-derived vesicles, this work bridges traditional herbal knowledge with advanced vesicle therapeutics, elucidating the osteogenic activity of PP-L-EVLPs and providing a theoretical foundation for developing Yunnan-specific medicinal plant resources. However, critical questions remain: The component–function relationships of PP-L-EVLPs require further investigation, particularly the relative contributions of its 1,439 lipid species and 739 proteins to osteoinduction. The specific intracellular pathways through which these bioactive components may synergistically drive osteogenic differentiation remain to be elucidated. Furthermore, future in vivo studies should employ large animal models (e.g., canine or porcine) with extended observation periods to rigorously validate the long-term stability and clinical translation potential of PP-L-EVLPs. Additionally, whether PP-L-EVLPs inherently home to periodontal defects or require functionalization for targeted therapy remains unclear.

**Fig. 7.**
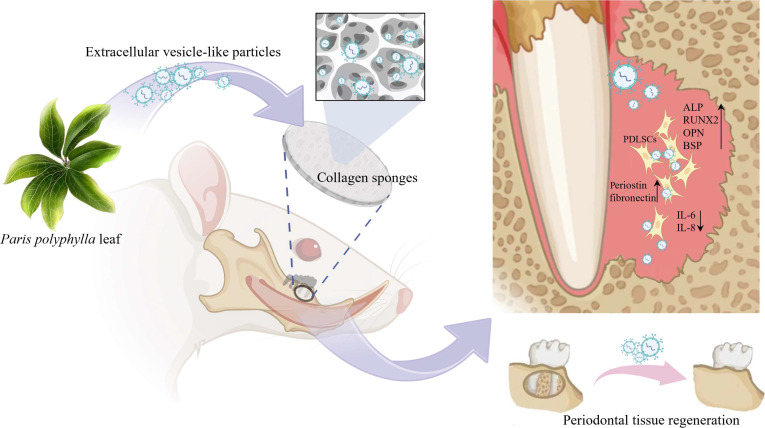
The mechanism underlying PP-L-EVLPs’ anti-inflammatory action and promotion of PDLSC osteogenic differentiation for effective regeneration of periodontal defect tissues.

In conclusion, this study demonstrates that PP-L-EVLPs, a novel plant-derived nanovesicle, effectively promote periodontal regeneration through migration-promoting, anti-inflammatory, and osteogenesis-enhancing activities (Fig. 7). Their biosafety and multi-component synergy provide a foundation for clinical translation. Future studies should focus on component–function relationships and targeted delivery strategies to optimize therapeutic efficacy.

## Data Availability

The datasets supporting the conclusions of this article are included within the article and its additional file.

## References

[1] Bawaskar HS, Bawaskar PH. Oral diseases: A global public health challenge. Lancet. 2020;395(10219):185–186. 10.1016/S0140-6736(19)33016-8 31954454

[2] Luo LS, Luan HH, Jiang JF, Wu L, Li C, Leng WD, Zeng XT. The spatial and temporal trends of severe periodontitis burden in Asia, 1990-2019: A population-based epidemiological study. J Periodontol. 2022;93(11):1615–1625. 35289931 10.1002/JPER.21-0625

[3] Kinane DF, Stathopoulou PG, Papapanou PN. Periodontal diseases. Nat Rev Dis Primers. 2017;22(3):17038. 10.1038/nrdp.2017.38 28805207

[4] Mahdizade Ari M, Amirmozafari N, Darbandi A, Afifirad R, Asadollahi P, Irajian G. Effectiveness of photodynamic therapy on the treatment of chronic periodontitis: A systematic review during 2008-2023. Front Chem. 2024;12:1384344. 38817441 10.3389/fchem.2024.1384344 PMC11138352

[5] Seo BM, Miura M, Gronthos S, Bartold PM, Batouli S, Brahim J, Young M, Robey PG, Wang CY, Shi S. Investigation of multipotent postnatal stem cells from human periodontal ligament. Lancet. 2004;364(9429):149–155. 15246727 10.1016/S0140-6736(04)16627-0

[6] Anvari Y, Afrashteh A, Pourkaveh S, Salek SB, Al-Numan L, Khademnezhad S. Emerging role of mesenchymal stem cell-derived extracellular vesicles in periodontal regeneration. J Taibah Univ Med Sci. 2024;19(2):390–402. 38380419 10.1016/j.jtumed.2024.01.006 PMC10876597

[7] Li Y, Zhou C, Liu H, Cai T, Fan H. Emerging roles of extracellular vesicles derived from bacteria, mammalian or plant cells in the pathogenesis and clinical application of neurodegenerative diseases. Biomolecules. 2024;14(3):312. 38540732 10.3390/biom14030312 PMC10968246

[8] Cui Y, Hong S, Xia Y, Li X, He X, Hu X, Li Y, Wang X, Lin K, Mao L. Melatonin engineering M2 macrophage-derived exosomes mediate endoplasmic reticulum stress and immune reprogramming for periodontitis therapy. Adv Sci. 2023;10(27): Article e2302029. 10.1002/advs.202302029 PMC10520618 37452425

[9] Hao S, Yang H, Hu J, Luo L, Yuan Y, Liu L. Bioactive compounds and biological functions of medicinal plant-derived extracellular vesicles. Pharmacol Res. 2024;200: Article 107062. 38211637 10.1016/j.phrs.2024.107062

[10] Cao M, Diao N, Cai X, Chen X, Xiao Y, Guo C, Chen D, Zhang X. Plant exosome nanovesicles (PENs): Green delivery platforms. Mater Horiz. 2023;10(10):3879–3894. 37671650 10.1039/d3mh01030a

[11] Sall IM, Flaviu TA. Plant and mammalian-derived extracellular vesicles: A new therapeutic approach for the future. Front Bioeng Biotechnol. 2023;11:1215650. 37781539 10.3389/fbioe.2023.1215650 PMC10534050

[12] Feng J, Xiu Q, Huang Y, Troyer Z, Li B, Zheng L. Plant-derived vesicle-like nanoparticles as promising biotherapeutic tools: Present and future. Adv Mater. 2023;35(24): Article e2207826. 36592157 10.1002/adma.202207826

[13] Zhang Z, Yu Y, Zhu G, Zeng L, Xu S, Cheng H, Ouyang Z, Chen J, Pathak JL, Wu L, et al. The emerging role of plant-derived exosomes-like nanoparticles in immune regulation and periodontitis treatment. Front Immunol. 2022;13: Article 896745. 35757759 10.3389/fimmu.2022.896745 PMC9231591

[14] Chen Y, Yan Q, Ji Y, Bai X, Li D, Mu R, Guo K, Yang M, Tao Y, Gershenzon J, et al. Unraveling the serial glycosylation in the biosynthesis of steroidal saponins in the medicinal plant *Paris polyphylla* and their antifungal action. Acta Pharm Sin B. 2023;13(11):4638–4654. 37969733 10.1016/j.apsb.2023.05.033 PMC10638507

[15] Ding YG, Zhao YL, Zhang J, Zuo ZT, Zhang QZ, Wang YZ. The traditional uses, phytochemistry, and pharmacological properties of Paris L. (Liliaceae): A review. J Ethnopharmacol. 2021;278: Article 114293. 34102270 10.1016/j.jep.2021.114293

[16] Guan L, Zheng Z, Guo Z, Xiao S, Liu T, Chen L, Gao H, Wang Z. Steroidal saponins from rhizome of Paris polyphylla var. chinensis and their anti-inflammatory, cytotoxic effects. Phytochemistry. 2024;219: Article 113994. 38244959 10.1016/j.phytochem.2024.113994

[17] Thapa CB, Paudel MR, Bhattarai HD, Pant KK, Devkota HP, Adhikari YP, Pant B. Bioactive secondary metabolites in *Paris polyphylla* Sm. And their biological activities: A review. Heliyon. 2022;8(2): Article e08982. 35243100 10.1016/j.heliyon.2022.e08982 PMC8881664

[18] Joshi B, Panda SK, Jouneghani RS, Liu M, Parajuli N, Leyssen P, Neyts J, Luyten W. Antibacterial, antifungal, antiviral, and anthelmintic activities of medicinal plants of Nepal selected based on ethnobotanical evidence. Evid Based Complement Alternat Med. 2020;2020:1043471. 32382275 10.1155/2020/1043471 PMC7193273

[19] Zhao Q, Wang T, Wang H, Cao P, Jiang C, Qiao H, Peng L, Lin X, Jiang Y, Jin H, et al. Consensus statement on research and application of Chinese herbal medicine derived extracellular vesicles-like particles (2023 edition). Chin Herb Med. 2023;16(1):3–12. 38375050 10.1016/j.chmed.2023.11.002 PMC10874762

[20] Stringer G. Chronic periodontitis, dantamoolaroga, indicates chronic systemic inflammation and reduces longevity. J Ayurveda Integr Med. 2024;15(6): Article 101048. 39626590 10.1016/j.jaim.2024.101048 PMC11647617

[21] Liu J, Ruan J, Weir MD, Ren K, Schneider A, Wang P, Oates TW, Chang X, Xu HHK. Periodontal bone-ligament-cementum regeneration via scaffolds and stem cells. Cells. 2019;8(6):537. 31167434 10.3390/cells8060537 PMC6628570

[22] Wei X, Xie H, Liu C, Li Y, Sun K, Qi B, Guo X, Liu Z, Huang X, Sun C, et al. Nature herbal medicine-tissue engineering strategies for regulate cell homeostasis in bone regeneration. Adv Funct Mater. 2025;35:2417810.

[23] Zhang J, Tian S, Guo L, Zhao H, Mao Z, Miao M. Chinese herbal medicine-derived extracellular vesicles as novel biotherapeutic tools: Present and future. J Transl Med. 2024;22(1):1059. 39587576 10.1186/s12967-024-05892-3 PMC11587639

[24] Zhang X, Gao H, Lin L. The extracellular vesicle-based treatment: A developing strategy for periodontal diseases. Front Immunol. 2025;29(16):1480292. 10.3389/fimmu.2025.1480292 PMC12158701 40510371

[25] Bachurski D, Schuldner M, Nguyen PH, Malz A, Reiners KS, Grenzi PC, Babatz F, Schauss AC, Hansen HP, Hallek M, et al. Extracellular vesicle measurements with nanoparticle tracking analysis—An accuracy and repeatability comparison between NanoSight NS300 and ZetaView. J Extracell Vesicles. 2019;8(1):1596016. 30988894 10.1080/20013078.2019.1596016 PMC6450530

[26] van Niel G, Carter DRF, Clayton A, Lambert DW, Raposo G, Vader P. Challenges and directions in studying cell-cell communication by extracellular vesicles. Nat Rev Mol Cell Biol*.* 2022;23(5):369–382. 35260831 10.1038/s41580-022-00460-3

[27] Khazaei F, Rezakhani L, Alizadeh M, Mahdavian E, Khazaei M. Exosomes and exosome-loaded scaffolds: Characterization and application in modern regenerative medicine. Tissue Cell. 2023;80: Article 102007. 36577349 10.1016/j.tice.2022.102007

[28] Kim MK, Choi YC, Cho SH, Choi JS, Cho YW. The antioxidant effect of small extracellular vesicles derived from *Aloe vera* peels for wound healing. Tissue Eng Regen Med. 2021;18(4):561–571. 34313971 10.1007/s13770-021-00367-8 PMC8325744

[29] Cao Y, Tan X, Shen J, Liu F, Xu Y, Chen Y, Zhou S, Qiu T, Li D, Zhao Q, et al. Morinda Officinalis-derived extracellular vesicle-like particles: Anti-osteoporosis effect by regulating MAPK signaling pathway. Phytomedicine. 2024;129: Article 155628. 38663117 10.1016/j.phymed.2024.155628

[30] Park YS, Kim HW, Hwang JH, Eom JY, Kim DH, Park J, Tae HJ, Lee S, Yoo JG, Kim JI, et al. Plum-derived exosome-like nanovesicles induce differentiation of osteoblasts and reduction of osteoclast activation. Nutrients. 2023;15(9):2107. 37432256 10.3390/nu15092107 PMC10180726

[31] Sim Y, Seo HJ, Kim DH, Lee SH, Kwon J, Kwun IS, Jung C, Kim JI, Lim JH, Kim DK, et al. The effect of apple-derived nanovesicles on the osteoblastogenesis of osteoblastic MC3T3-E1 cells. J Med Food. 2023;26(1):49–58. 36594993 10.1089/jmf.2022.K.0094

[32] Huang G, Xia B, Dai Z, Yang R, Chen R, Yang H. Comparative study of dedifferentiated fat cell and adipose-derived stromal cell sheets for periodontal tissue regeneration: In vivo and in vitro evidence. J Clin Periodontol. 2022;49(12):1289–1303. 35851962 10.1111/jcpe.13705

[33] Liang L, Wang L, Liao Z, Ma L, Wang P, Zhao J, Wu J, Yang H. High-yield nanovesicles extruded from dental follicle stem cells promote the regeneration of periodontal tissues as an alternative of exosomes. J Clin Periodontol. 2024;51(10):1395–1407. 38951121 10.1111/jcpe.14036

[34] Ma L, Rao N, Jiang H, Dai Y, Yang S, Yang H, Hu J. Small extracellular vesicles from dental follicle stem cells provide biochemical cues for periodontal tissue regeneration. Stem Cell Res Ther. 2022;13(1):92. 35241181 10.1186/s13287-022-02767-6 PMC8895915

[35] Zhao Q, Feng J, Liu F, Liang Q, Xie M, Dong J, Zou Y, Ye J, Liu G, Cao Y, et al. Rhizoma Drynariae-derived nanovesicles reverse osteoporosis by potentiating osteogenic differentiation of human bone marrow mesenchymal stem cells via targeting ERα signaling. Acta Pharm Sin B. 2024;14(5):2210–2227. 38799625 10.1016/j.apsb.2024.02.005 PMC11119514

[36] Zhan W, Deng M, Huang X, Xie D, Gao X, Chen J, Shi Z, Lu J, Lin H, Li P. Pueraria lobata-derived exosome-like nanovesicles alleviate osteoporosis by enhacning autophagy. J Control Release. 2023;364:644–653. 37967723 10.1016/j.jconrel.2023.11.020

[37] Sundaram K, Miller DP, Kumar A, Teng Y, Sayed M, Mu J, Lei C, Sriwastva MK, Zhang L, Yan J, et al. Plant-derived exosomal nanoparticles inhibit pathogenicity of porphyromonas gingivalis. iScience. 2020;23(2): Article 100869. 10.1016/j.isci.2020.100869 PMC7054809 32058949

