# Supplementary material for: Paris polyphylla var. yunnanensis Leaf-Derived Extracellular Vesicle-Like Particles Enhance Periodontal Regeneration
Source: Biomater Res. 2025 Dec 9;29:0291. doi: 10.34133/bmr.0291 (PMC12688648; doi:10.34133/bmr.0291)
Supplement: Supplementary 1 — Figs. S1 to S6 Table S1 [file bmr.0291.f1.zip › Supporting Information.docx]

Fig. S1 Isolation, identification of PDLSCs. (A) Primary cultured of PDLSCs. Scale bar: 100 μm. (B) PDLSC cell colonies. Scale bar: 200 μm. (C) Alizarin red staining of PDLSCs after 21 days of osteogenic induction. Scale bar: 50 μm. (D) Oil red O staining of PDLSCs after 21 days of adipogenic induction. Scale bar: 50 μm (E) PDLSCs detected by flow cytometry. Cells are positive for CD44, CD29, CD90 and CD105, but are negative for CD34 and CD45.

Fig. S2 Uptake of PP-L-EVLPs by PDLSCs. PDLSCs were co-incubated with PP-L-EVLPs, which were labeled with the red fluorescent dye Dil, and observed using confocal laser scanning microscopy. A: Bright-field image showing the morphology of PDLSCs. B: Nuclei were stained with Hoechst (blue).C: Fluorescence image showing the signal (red) from DiI-labeled extracellular vesicles. D: Merged image reveals that the vesicle signals (red) are located within the cytoplasm, indicating successful uptake of the extracellular vesicles by PDLSCs. Scale bar: 10μm.

Fig. S3 PP-L-EVLPs promote osteogenic differentiation of PDLSCs *in vitro*. (A) Alkaline phosphatase (ALP) staining images after 3 days of PP-L-EVLP induction(scale bar =100μm). (B) ARS images of PDLSCs on day 7 osteogenic differentiation(scale bar =100μm). (C) Quantitative analysis of alkaline phosphatase (ALP) and Alizarin Red S staining(n=3). (D) Effect of PP-L-EVLPs on osteogenesis-related markers mRNA levels in PDLSCs on day 7 of osteogenesis differentiation was detected by PCR. Data are presented as mean ±SD (n=3). **p<0.05, **p<0.01, ***p<0.001, ****p<0.0001.*

Fig. S4 Safety evaluation of PP-L-EVLPs *in vivo*. (A) H&E staining of different organs showed no obvious histological changes after 2, 4 week of treatment. Scale bar = 100 μm. (B) Immunohistochemistry staining was used to observe the expression of γ-H2A.X. Scale bar = 200 μm.

Fig. S5 Live/Dead staining of PDLSCs treated with PP-L-EVLPs. PDLSCs were incubated with the different concentrations of PP-L-EVLPs for 24 hours. Live cells (stained with calcein-AM) are shown in green, and dead cells (stained with propidium iodide) are shown in red. Scale bar: 100 μm.

Fig. S6 Macrophage polarization in rat periodontal defects by immunofluorescence staining. Representative images of (A) iNOS (M1 macrophage marker) and (B) CD163 (M2 macrophage marker) immunofluorescence in periodontal defect areas at 2, 4, and 6, weeks post-surgery. Nuclei were counterstained with DAPI (blue). The images demonstrate a shift in macrophage phenotype from early M1 dominance to a sustained M2 predominance in the PP-L-EVLPs treatment group over time. Low-magnification (scale bar: 500 μm); high-magnification (scale bar: 100 μm).

Table S1 Primers sequence for reverse transcription-quantitative polymerase chain reaction

| Gene | Forward primer (5’-3’) | Reverse primer (5’ -3’) |
| --- | --- | --- |
| *ALP* | GCCTACACGGTCCTCCTATACG | TGCTGACTGCTGCCGATACTC |
| *Runx2* | AGGCAGTTCCCAAGCATTTCATC | AGTGAGTGGTGGCGGACATAC |
| *OPN* | TGGAAAGCGAGGAGTTGAATGGTG | AATCTGGACTGCTTGTGGCTGTG |
