# Supplementary material for: Paris polyphylla var. yunnanensis Leaf-Derived Extracellular Vesicle-Like Particles Enhance Periodontal Regeneration
Source: Biomater Res. 2025 Dec 9;29:0291. doi: 10.34133/bmr.0291 (PMC12688648; doi:10.34133/bmr.0291)
Supplement: Supplementary 1 — Figs. S1 to S6 Table S1 [file bmr.0291.f1.zip › Table S1.docx]

Table S1 Primers sequence for reverse transcription-quantitative polymerase chain reaction

| Gene | Forward primer (5’-3’) | Reverse primer (5’ -3’) |
| --- | --- | --- |
| *ALP* | GCCTACACGGTCCTCCTATACG | TGCTGACTGCTGCCGATACTC |
| *Runx2* | AGGCAGTTCCCAAGCATTTCATC | AGTGAGTGGTGGCGGACATAC |
| *OPN* | TGGAAAGCGAGGAGTTGAATGGTG | AATCTGGACTGCTTGTGGCTGTG |
