# Supplementary figures and images for: Paris polyphylla var. yunnanensis Leaf-Derived Extracellular Vesicle-Like Particles Enhance Periodontal Regeneration
Source: Biomater Res. 2025 Dec 9;29:0291. doi: 10.34133/bmr.0291 (PMC12688648; doi:10.34133/bmr.0291)

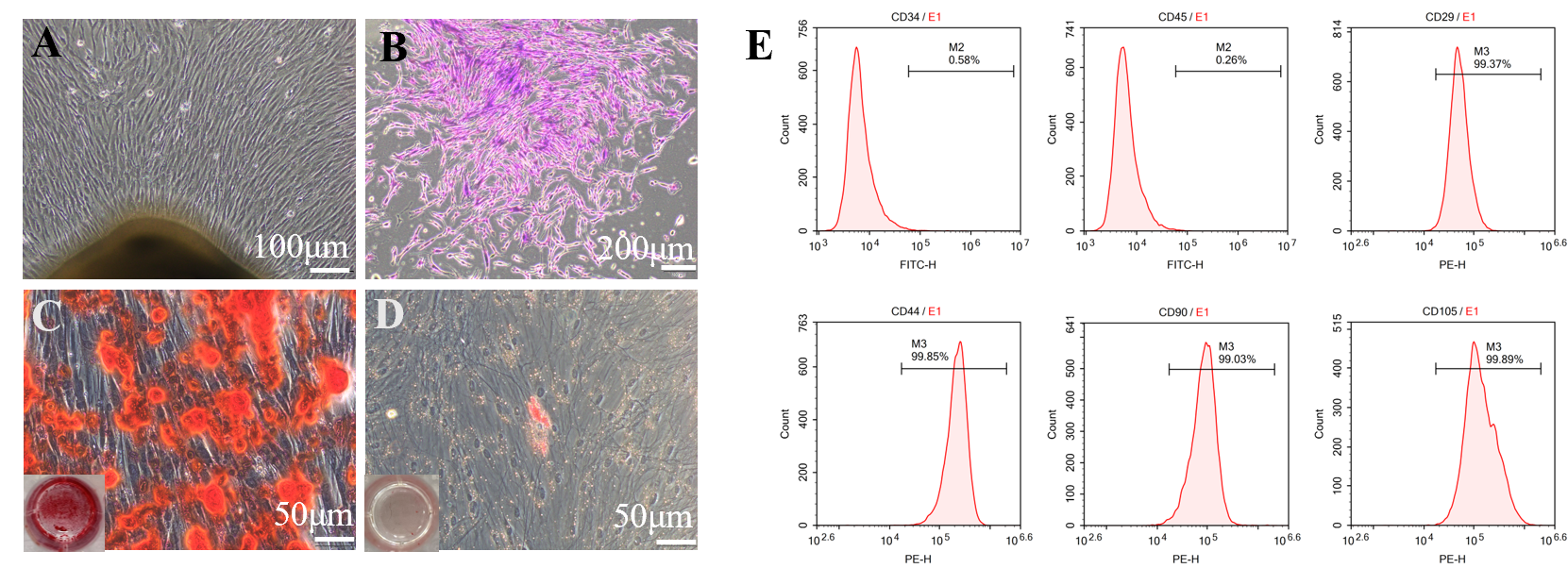

Supplement: Supplementary 1 — Figs. S1 to S6 Table S1 [file bmr.0291.f1.zip › figure S1.tif]

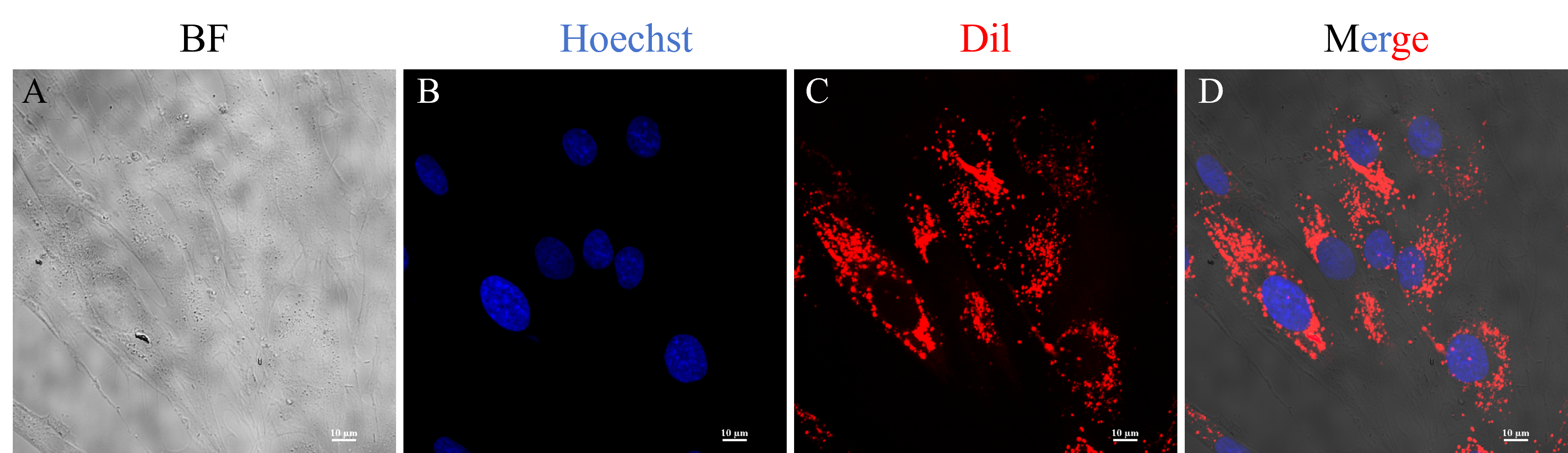

Supplement: Supplementary 1 — Figs. S1 to S6 Table S1 [file bmr.0291.f1.zip › Figure S2 .tif]

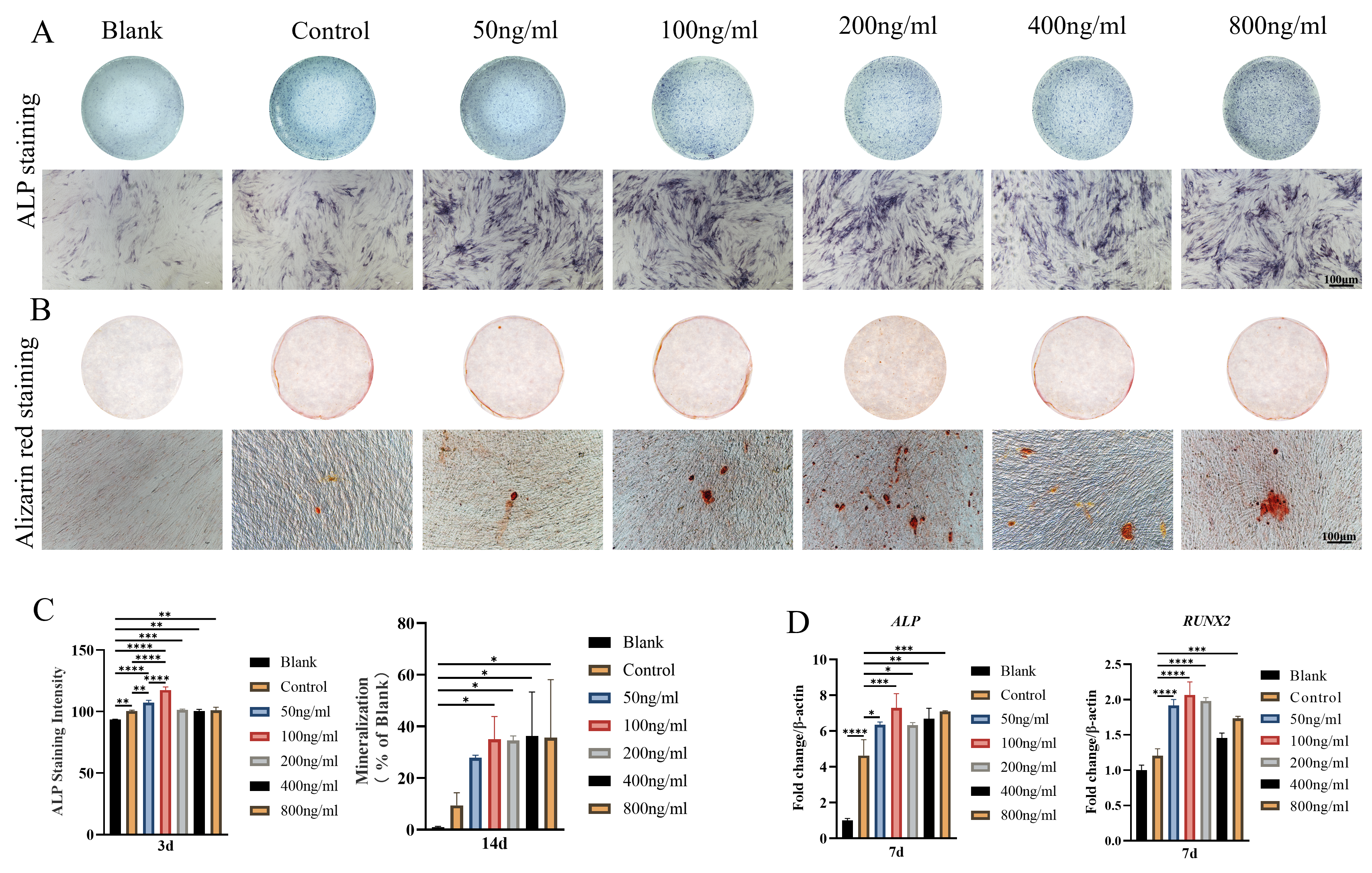

Supplement: Supplementary 1 — Figs. S1 to S6 Table S1 [file bmr.0291.f1.zip › Figure S3 .tif]

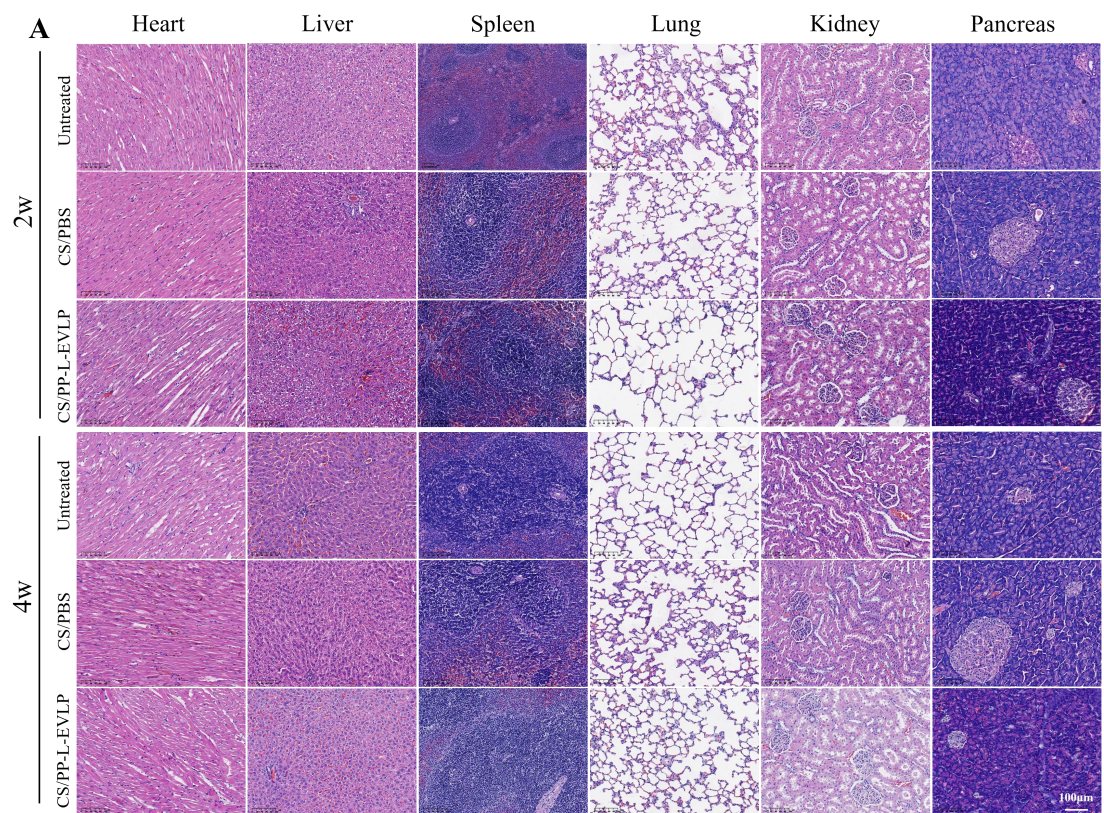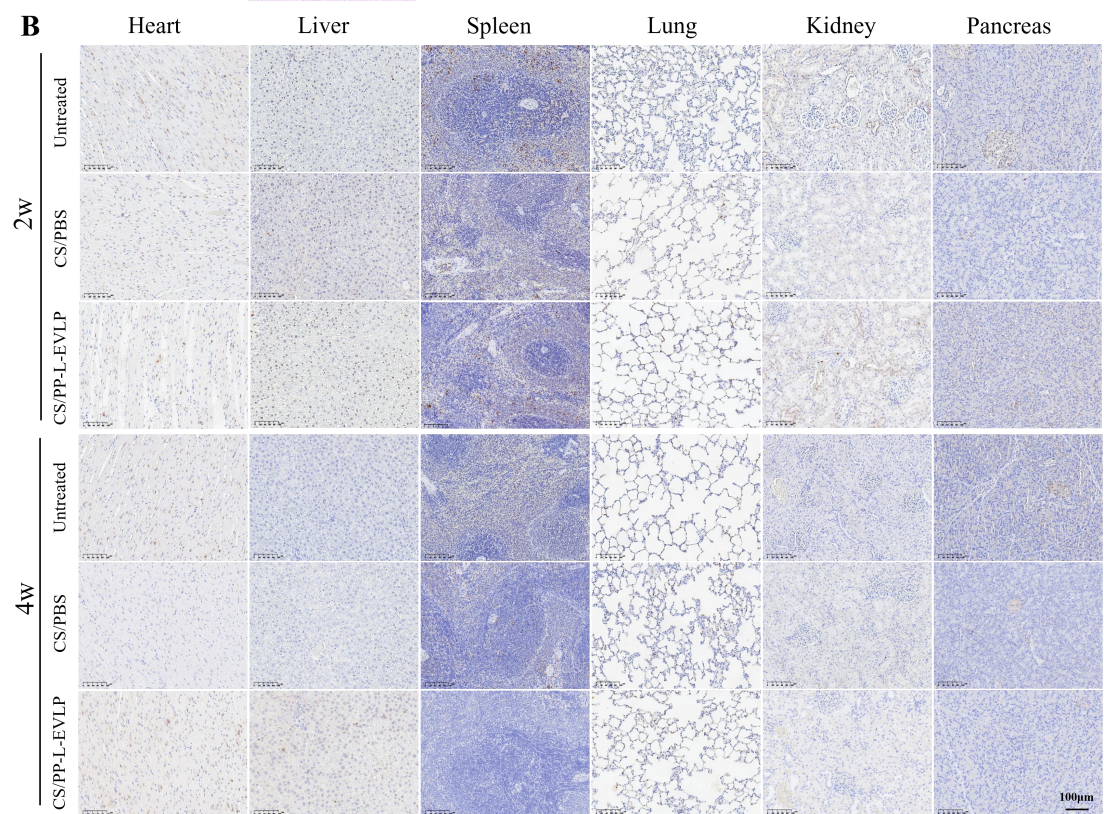

Supplement: Supplementary 1 — Figs. S1 to S6 Table S1 [file bmr.0291.f1.zip › Figure S4.pdf]

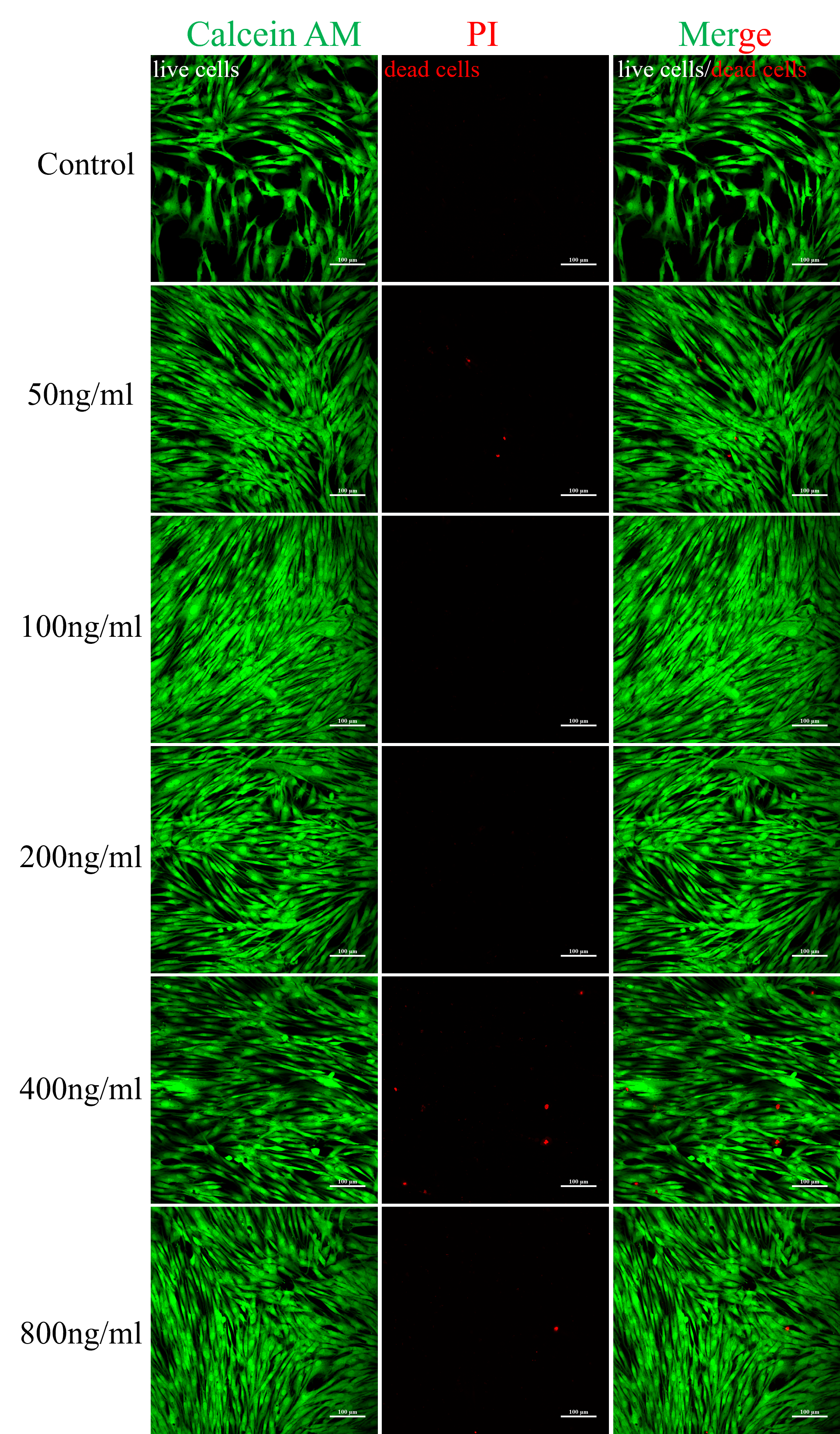

Supplement: Supplementary 1 — Figs. S1 to S6 Table S1 [file bmr.0291.f1.zip › Figure S5.tif]

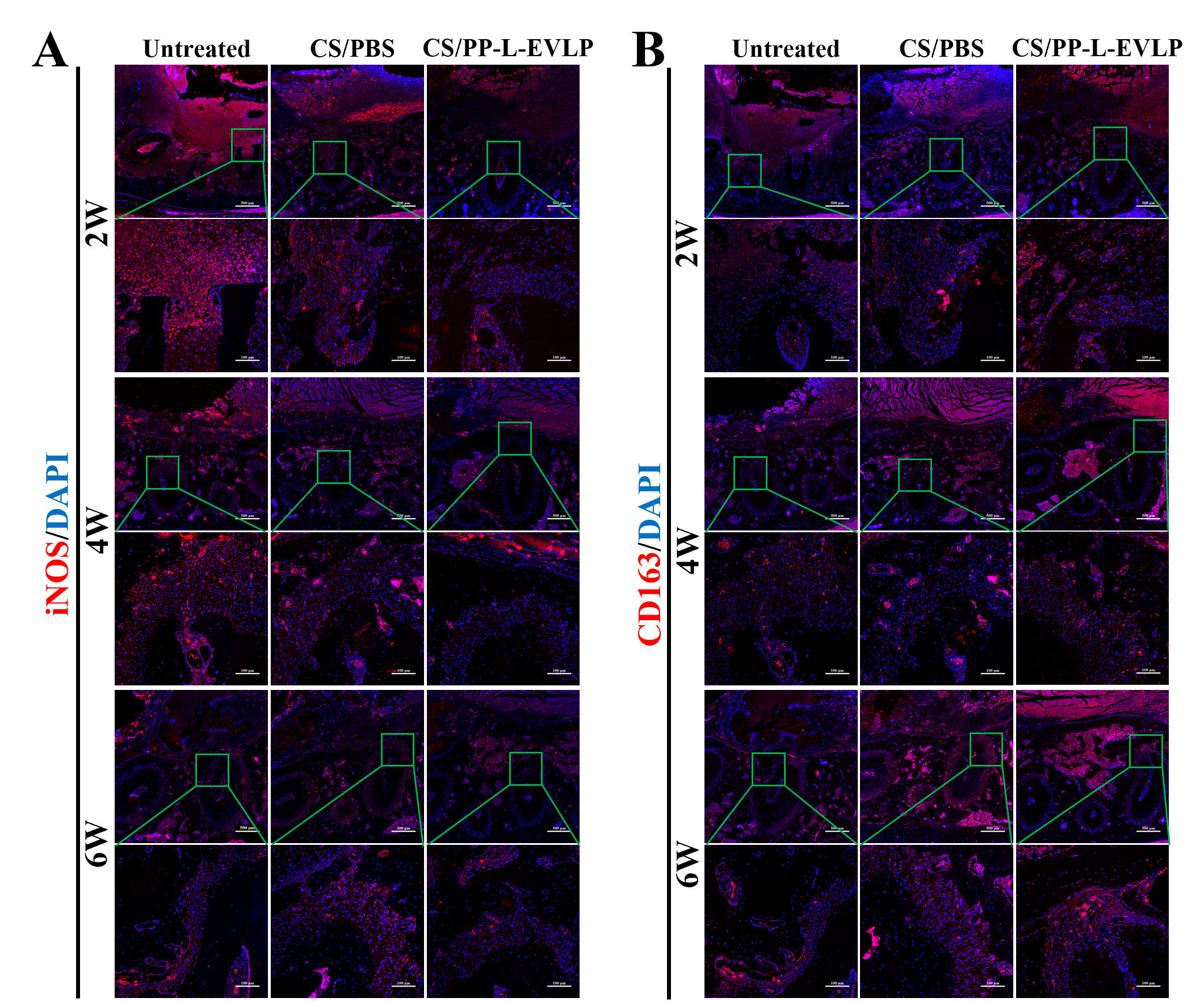

Supplement: Supplementary 1 — Figs. S1 to S6 Table S1 [file bmr.0291.f1.zip › Figure S6.tif]
